# Dataset on genetic and physiological adults׳ responses to social distress

**DOI:** 10.1016/j.dib.2017.06.057

**Published:** 2017-07-05

**Authors:** Andrea Bonassi, Tommaso Ghilardi, Anna Truzzi, Ilaria Cataldo, Atiqah Azhari, Peipei Setoh, Kazuyuki Shinohara, Gianluca Esposito

**Affiliations:** a Department of Psychology and Cognitive Science, University of Trento, Rovereto, Italy; b Affiliative and Social Behavior Laboratory, Brain Science Institute, RIKEN, Saitama, Japan; c Division of Psychology, School of Humanities and Social Sciences, Nanyang Technological University, Singapore; d Department of Neurology and Behavior, School of Medicine, Nagasaki University, Nagasaki, Japan

**Keywords:** Adult interaction, Oxytocin receptor gene, Social abilities, Gene*environment, Physiological responses to social distress

## Abstract

Both expectations towards interactions with conspecifics, and genetic predispositions, affect adults׳ social behaviors. However, the underlying mechanisms remain largely unknown. Here, we report data to investigate the interaction between genetic factors, (oxytocin receptor (OXTR) and serotonin transporter (5-HTTLPR) polymorphisms), and adult interactional patterns in shaping physiological responses to social distress. During the presentation of distress vocalizations (cries of human female, infants and bonobos) we assessed participants׳ (*N* = 42 males) heart rate (HR) and peripheral nose temperature, which index state of arousal and readiness to action. Self-reported questionnaires were used to evaluate participants’ interactional patterns towards peers (Attachment Style Questionnaire, Feeney et al., 1994[1]), and the quality of bond with intimate partners (Experiences in Close Relationships Scale, Fraley et al., 2000 [2]). To assess participants׳ genetic predispositions, the OXTR gene (regions rs53576, and rs2254298) and the 5-HTTLPR gene (region SLC6A4) were genotyped. The data set is made publicly available to enable critical or extended analyzes.

**Specifications Table**

**Table:** 

Subject area	*Psychology*
More specific subject area	*Psychobiology – Psychophysiology – Behavioral Psychology – Developmental Psychology*
Type of data	*Text file, Figure*
How data was acquired	*Flex Comp Infiniti Thought Technology Ltd.* *Applent at4524 multi-channel temperature meter* *NanoDrop Technologies, USA* *Applied Biosystems, Inc.*
Data format	*Pre-processed (averages per condition), Analyzed*
Experimental factors	– *Characteristics of interpersonal relationships assessed with self reported questionnaires (ASQ: 4 subscales; ECR-R: 2 subscales)* – *Three genetic factors (three polymorphic regions: two on the oxytocin receptor gene, one on the serotonin transporter gene)*
Experimental features	*Participants׳ physiological responses (HR, NT) were measured during the presentation of distress stimuli. Consecutively, self reported questionnaires (ASQ, ECR-R) and genetic predispositions (OXTR gene׳s rs53576 and rs2254298 regions, 5-HTTLPR gene) were assessed to investigate how social expectation and gene factor interact by shaping physiological activations.*
Data source location	*Rovereto (TN), Italy, 45.892351, 11.043844*
Data accessibility	Supplementary materials

**Value of data**

• This study provides data to gain a gene×environment perspective on how adults׳ responses to social distress events are shaped by subjective innate predispositions and individuals׳ social experience.
• These data are potentially useful to investigate the underlying mechanisms that lead to individuals׳ differential physiological responses to social distress.
• These data are potentially useful to investigators studying adaptive responses to social distress from a multilevel perspective.

## 1 Data

Conspecific interactions constitute a fundamental basis of human sociality. Phylogenetically, social interactions increase human survival [3], [4], [5], [6]; ontogenetically, social interactions possess evolutionary value as they shape the development of cognitive, social, and emotional abilities [7]. Phylogenesis and ontogenesis converge when social interaction is utilized as an avenue to foster cooperation, form relationships with partners and facilitate reproduction. Social experience evolves from early interactions with parents since the first year of life. These experiences continue to influence the course of social development of a human being, and eventually becomes expressed in our relations to peers and partners in adulthood.

Although adult social interaction with people and intimate partners are indubitably influenced by the environment, each individual׳s unique characteristics remain indispensable in this process. For this reason, a multi-level approach that simultaneously investigates genetic predisposition and environmental-level experiential factors is preferable to analyze how social sensitivity develops [8], [9].

**Fig. 1:**
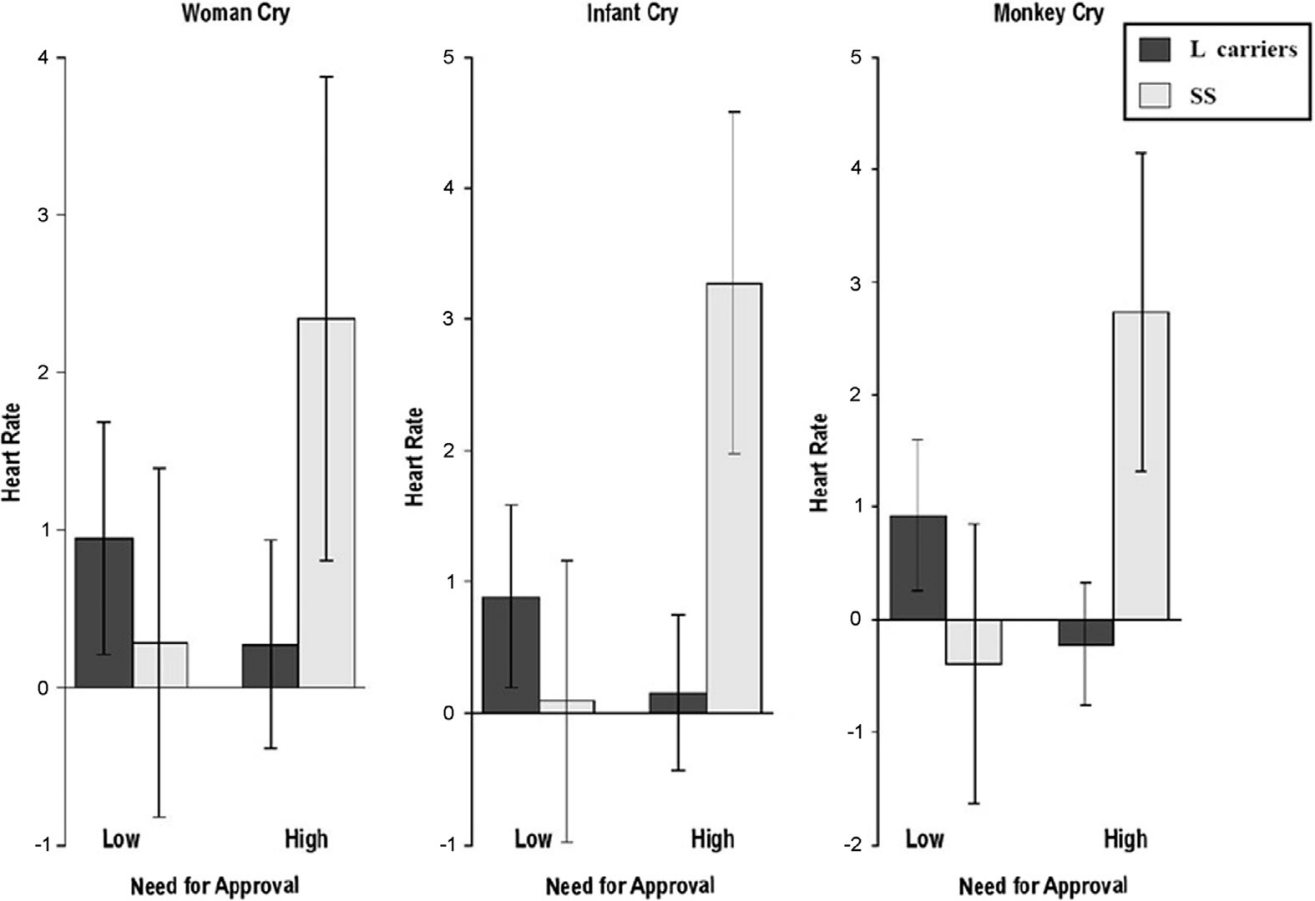
Need for Approval x Cries x 5-HTTLPR.

**Fig. 2:**
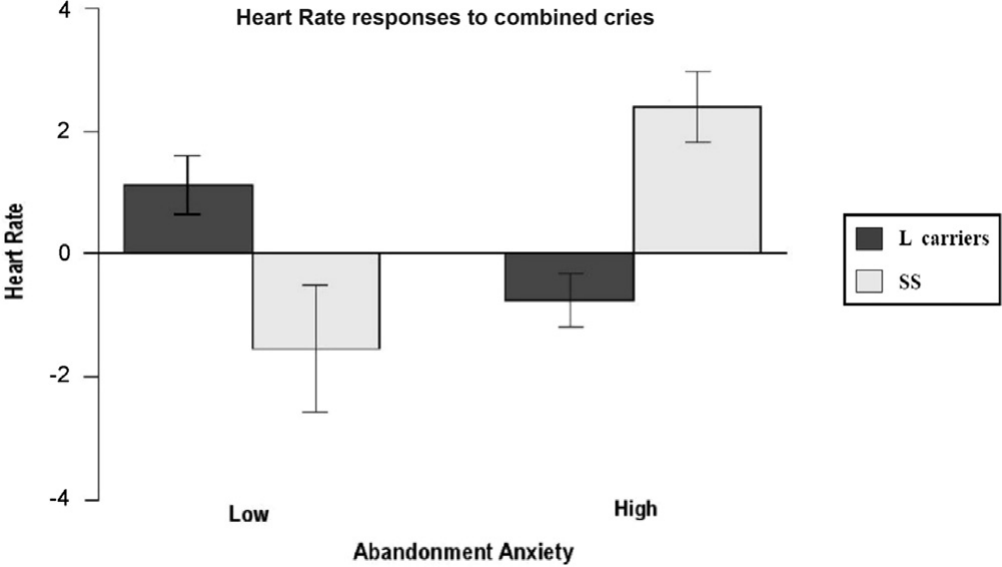
Abandonment Anxiety x 5-HTTLPR.

**Fig. 3:**
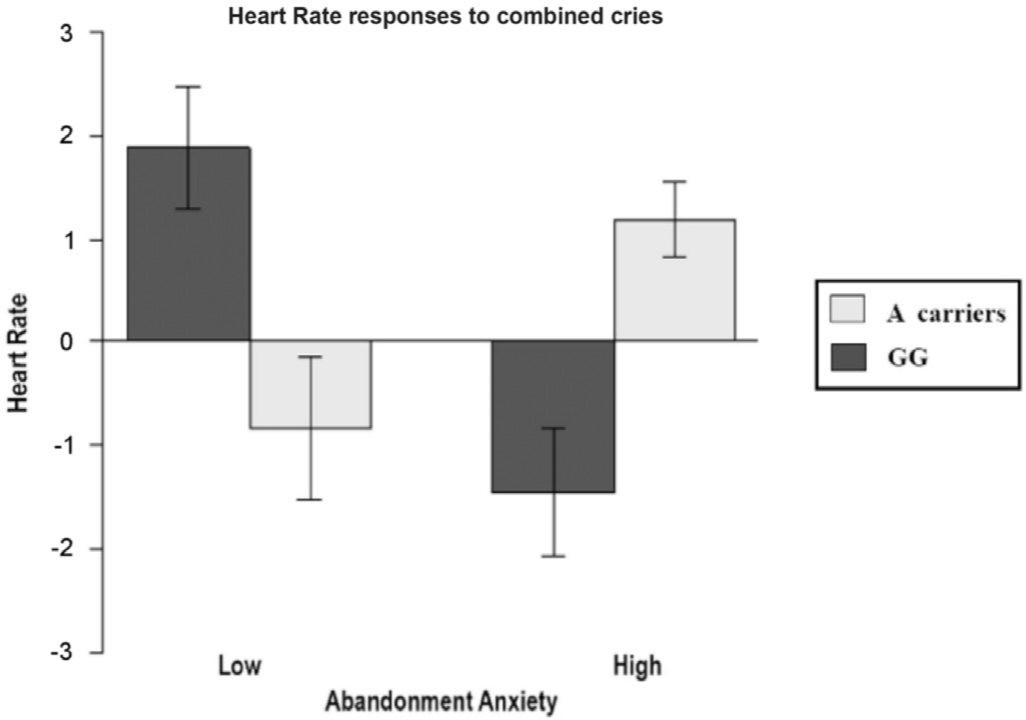
Abandonment Anxiety x rs53576.

Participants completed two online self-report questionnaires to assess their attachment status towards peers and partners (ASQ: Attachment Style Questionnaire and ECR-R: Experiences in Close Relationships Scale). After the questionnaires have been completed, participants attend the experimental session. Heart rate and nasal peripheral temperature were recorded throughout the presentation of the 30 distressing audio clips. At the end of each experimental session, a buccal mucosa sample was collected from each participant. Fig. 1, Fig. 2, Fig. 3 report averages physiological responses of participants accordingly to their genetic characteristics and attachment status. Data are available in the Supplementary materials.

In this figure Gene*Environment*Stimuli interaction between the ASQ dimension *Need for Approval*, cries׳ categories 5-HTTLPR genotype over HR responses are reported.

Here the interaction between the ECR-R dimension *Abandonment Anxiety* 5-HTTLPR genotype over HR responses is reported. Here Heart Rate variation in response to all combined cries are represented.

Here Heart Rate variation in response to all combined cries are represented.

In Fig. 3 the interaction between the ECR-R dimension *Abandonment Anxiety* and rs53576 genotype is presented.

## 2 Experimental design, materials and methods

### 2.1 Participants

Forty-two non-parent adult males (*M*=24.7 years, *SD*=5.05) were recruited through a database of volunteers made available on the University of Trento website. Informed consent was obtained from all participants, and the study was conducted in accord with ethical principles stated in the Helsinki declaration.

### 2.2 Stimuli

The stimuli were 30 15-s audio clips of distressed vocalizations, ten clips for each of 3 categories: infant cries, adult female cries, and bonobo cries. Cries were chosen because of their evolutionary significance and have been found to elicit distress and specific physiological responses in adults [10], [11], [12].

This study aimed to assess physiological responses elicited by social distress. Since infant and female cries possess a specific evolutionary salience to male adults, these cries were included as stimuli. Also, to investigate whether the physiological responses elicited were specific for human distressed vocalizations, or a generalized response to social distress, bonobo cries were included in the stimulus set.

All cry stimuli were normalized for intensity, and the volume was kept constant throughout all stimuli presentations. Each audio clip was presented following 10 s of silence. Audio clips were organized into three different randomized sequences, and presentation order of the three sequences was counterbalanced across participants. Stimulus sequences were created using open source software Audacity.

### 2.3 Attachment Style Questionnaire (ASQ)

The ASQ [1] is a 40-item self-report questionnaire developed to measure five variables (*Confidence, Fear of Intimacy, Relationship as Secondary, Need for Approval, Preoccupation with Relationship*) that determine individual differences in adult attachment. *Confidence* measures the level of self-assurance (i.e. *Overall, I am a worthwhile person*). *Fear of Intimacy* measures discomfort in intimate situations (i.e. *I prefer to depend on myself*). *Relationship as Secondary* measures the superficiality of a relationship (i.e. *To ask for help is to admit*). *Need for Approval* measures the need to be validated by others (i.e. *It׳s important to me that others like me*). *Preoccupation with Relationship* measures the extent of attachment anxiety in a relationship (i.e. *I find that others are reluctant*) [13].

### 2.4 Experience in Close Relationships – Revised (ECR-R)

The ECR-R [2] is a 36-item self-report questionnaire developed to measure the degree to which two variables (Anxiety, Avoidance) are present in a relationship with an intimate partner. The result is indicative of the individual׳s attachment status towards the partner. Anxiety measures jealousy and fear of abandonment (i.e. *I’m afraid that I will lose my partner׳s love*). Avoidance measures the tendency to refrain from intimacy (i.e. *I prefer not to be too close to romantic partners*) [14], [15].

### 2.5 Heart rate

HR was measured to assess participants’ arousal and stressful/calming states. An increase in heart rate indices underline an increase in attention and promptness to action, whereas a decrease reflects the activation of a calming reaction in response to external stimulation [16], [17].

### 2.6 Temperature

To index sympathetic activity, we measured participants’ peripheral surface body temperature at the tip of the nose using a thermistor (applent at 4524 multi-channel temperature meter) throughout the duration of the experiment. Temperature was measured to assess participants’ promptness to action and perception of the emotional quality of the stimuli. Changes in temperature at this extremity reflect a recognition of emotional valence of the stimuli and an increase in arousal. Increases in facial temperature suggest the presence of positive external stimuli, while a reduction in temperature indicate the occurrence of negative external stimuli [18], [19].

### 2.7 Genetic assessment

DNA extraction and genotyping were conducted by ACGT,Inc. (Wheeling, IL). DNA was extracted from each kit using the Oragene DNA purification reagent. DNA concentrations were evaluated using spectroscopy (NanoDrop Technologies, USA). Each DNA sample was polymerase chain reaction (PCR) amplified for the rs2254298 region target with the primers 5-TGA AAG CAG AGG TTG TGT GGA CAG G-3 and 5-AAC GCC CAC CCC AGT TTC TTC-3. A PCR reaction of 20 ll consisting of 1.5 ll of genomic DNA from the test sample, PCR buffer, 1 mM each of forward and reverse primers, 10 mM deoxyribonucleotides, KapaTaq polymerase, and 50 mM MgCl2 was performed. Cycling conditions included an initial 15 min denaturation at 95 °C, and 35 cycles of 94 °C (30 s), 60 °C(60 s), 72 °C (60 s), and a final extension of 72 °C for 10 min. PCR reactions were genotyped with an ABI 3730xl Genetic Analyzer (Applied Biosystems Inc.) and normalized with GeneScan 600 LIZ (Applied Biosystems, Inc.) size standards run on each sample. Genotypic data was analyzed using GeneMapper ID (Applied Biosystems, Inc.). Participants possessing at least one A allele (A/A or G/A) were classified into a single A carriers group. In the general population, the distribution of different genotypes for this DNA region is 79% for G/G homozygotes and 21% for A carriers.

Similar DNA extraction procedures were applied for rs53576. However, the forward and reverse primers that were used were: 5′-GCCCACCATGCTCTCCACATC-3′ and 5′-GCTGGACTCAGGAGGAATAGGGAC-3′. Participants possessing at least one A allele (A/A or G/A) were classified into a single A carriers group. In the general population, the distribution of different genotypes for this DNA region is 61% for G/G homozygotes and 39% for A carriers.

For the 5-HTTLPR gene (region SLC6A4), each DNA sample was polymerase chain reaction (PCR) amplified with a forward primer (50-CCAGCACCTAACCCCTAAT-30) labeled with a fluorescent dye, 6-carboxyfluorescein (6-FAM), and a reverse primer (50-AGGGACTGAGCTGGACAACCAC-30). The same DNA extraction optimization conditions and sequencing procedures were used. Participants possessing at least one L allele (L/L or L/S) were classified into a single L carriers group (*N*=30), while S/S homozygotes were considered as a second group (*N*=12).

### 2.8 Preliminary analysis

The average response of each physiological parameter was calculated for every stimulus presentation, after which residuals were calculated from the baseline position. The different distress sounds (human adult female, human infant, bonobo) were considered separately to test for participants’ reactions to three distinct categories of distress vocalizations. For every subject, an average HR and peripheral body temperature value, in response to each of the three different sounds, was calculated. For each questionnaire dimension, participants’ were divided into two groups, high and low, applying the median split procedure and this factorial measure was then used in subsequent analysis. Twelve multivariate ANOVAs were then performed, one for each physiological variable (HR, nose temperature), each single nucleotide polymorphism considered (rs53576, rs2254298, SLC6A4) and each questionnaire (ECR, ASQ). In the ANOVAs the physiological values were used as dependent variable, the distress type (infant, woman, bonobo) as within-subject factor, and the genotype and the questionnaires’ dimensions as between-subject factors. As post-hoc analysis, t-student tests were run to verify differences in physiological responses between groups.

## Footnotes

Appendix A Supplementary data associated with this article can be found in the online version at 10.1016/j.dib.2017.06.057.
