# Supplementary material for: Dataset on genetic and physiological adults׳ responses to social distress
Source: Data Brief. 2017 Jul 5;13:742–8. doi: 10.1016/j.dib.2017.06.057 (PMC5512186; doi:10.1016/j.dib.2017.06.057)
Supplement: Supplementary file 1 — Supplementary material [file mmc1.docx]

Rovereto,Trento (Italy) February 27^th^ 2017

Dear Editor-in-Chief Dr. Hao-Ran Wang

Please find attached our manuscript titled: “**Genetic factors and social expectations interact in affecting physiological responses to social distress**”. The authors of this paper are Andrea Bonassi, Tommaso Ghilardi, Anna Truzzi, Ilaria Cataldo, Atiqah Azhari, Kazuyuki Shinohara, and Gianluca Esposito. The manuscript has been read and approved by all authors. The paper is not under consideration for publication elsewhere, and it has been approved by all authors and tacitly by the responsible authorities where the work was carried out, and that, if accepted, it will not be published elsewhere in the same form, in English or in any other language, including electronically without the written consent of the copyright-holder. In this paper, we present a study about the long-term effect of gene * environment interaction on individuals’ physiological responses to social distress. The study has been conducted in accordance with ethical principles stated in the Helsinki declaration. Informed consent was obtained from all participants. The genetic assessment was conducted on anonymized bio-samples at the University of Nagasaki (Japan), and followed the procedures approved by the IRB guidelines of the University of Nagasaki Graduate School Of Medicine. Authors declare no conflict of interest.

The data will be available upon request to the corresponding author.

We hope you can consider our paper for publication in ***Data in Brief.***

Best regards,

Gianluca Esposito, Ph.D

University of Trento (Italy)

Nanyang Technological University (Singapore)
